# Biological characteristics of intratumoral [F-18]-fluoromisonidazole distribution in a rodent model of glioma

**DOI:** 10.3892/ijo.2013.1781

**Published:** 2013-01-18

**Authors:** TOSHIYUKI HATANO, SONGJI ZHAO, YAN ZHAO, KEN-ICHI NISHIJIMA, NORIHITO KUNO, HIROKO HANZAWA, TAKESHI SAKAMOTO, NAGARA TAMAKI, YUJI KUGE

**Affiliations:** 1 Central Institute of Isotope Science, Hokkaido University, Sapporo;; 2 Central Research Laboratory, Hitachi Ltd., Kokubunji, Tokyo;; 3 Departments of Tracer Kinetics and Bioanalysis, Graduate School of Medicine, Hokkaido University, Sapporo, Japan; 4 Nuclear Medicine, Graduate School of Medicine, Hokkaido University, Sapporo, Japan

**Keywords:** [F-18]-fluoromisonidazole, tumor hypoxia, cellular proliferation, glucose metabolism

## Abstract

Accurate imaging to identify hypoxic regions in tumors is key for radiotherapy planning. [F-18]-fluoromisonidazole ([F-18]-FMISO) is widely used for tumor hypoxia imaging and has the potential to optimize radio-therapy planning. However, the biological characteristics of intratumoral [F-18]-FMISO distribution have not yet been fully investigated. In hypoxic cells, the hypoxia-inducible factor-1 (HIF-1) target proteins that induce cellular prolif-HIF-1) target proteins that induce cellular proliferation and glucose metabolism, glucose transporter-1 (Glut-1) and hexokinase-II (HK-II), are upregulated. In this study, we determined the intratumoral distribution of [F-18]-FMISO by autoradiography (ARG) and compared it with pimonidazole uptake, expression of Glut-1, tumor proliferative activity (Ki-67 index) and glucose metabolism ([C-14]2-fluoro-2-deoxy-D-glucose uptake; [C-14]-FDG) in a glioma rat model. Five C6 glioma-bearing rats were injected with [F-18]-FMISO and [C-14]-FDG. After 90 min, the rats were injected with pimonidazole and 60 min later, the rats were sacrificed and tumor tissues were sectioned into slices. The adjacent slices were used for ARG and immunohistochemical (IHC) analyses of pimonidazole, Glut-1 and Ki-67. [F-18]-FMISO ARG images were divided into regions of high [F-18]-FMISO uptake (FMISO+) and low [F-18]-FMISO uptake (FMISO−). Pimonidazole and Glut-1 expression levels, Ki-67 index and [C-14]-FDG distribution were evaluated in the regions of interest (ROIs) placed on FMISO+ and FMISO−. [F-18]-FMISO distribution was generally consistent with pimonidazole distribution. The percentage of positively stained areas (% positive) of Glut-1 in FMISO+ was significantly higher compared to FMISO (24±8% in FMISO+ and 9±4% in FMISO−; P<0.05). There were no significant differences in Ki-67 index and [C-14]-FDG uptake between FMISO+ and FMISO− (for Ki-67, 10±5% in FMISO+ and 12±5% in FMISO−, P = ns; for [C-14]-FDG, 1.4±0.3% ID/g/kg in FMISO+ and 1.3±0.3% ID/g/kg in FMISO−, P = ns). Intratumoral [F-18]-FMISO distribution reflected tumor hypoxia and expression of the hypoxia-related gene product Glut-1; it did not, however, reflect tumor proliferation or glucose metabolism. Our findings help elucidate the biological characteristics of intratumoral [F-18]-FMISO distribution that are relevant to radiotherapy planning.

## Introduction

Hypoxia develops in solid tumors due to the insufficiency of oxygen diffusion (chronic hypoxia) and blood perfusion (acute hypoxia) (1). Chronic hypoxia is the result of the long distance of tumor cells from the nearest blood vessel. Acute hypoxia is the result of fluctuating flow in blood vessels. Tumor cells in a hypoxic environment are more resistant to radiation damage than those in a normal oxygen environment, since radiation damage sensitivity depends on the oxygen concentration at the time of irradiation (2). Hypoxic regions are also related to tumor malignancy and proliferation (3–5). Therefore, hypoxia imaging can provide useful information for radiotherapy planning, including intensity-modulated radiation therapy (IMRT), and may also be a useful prognostic tool. [F-18]-Fluoromisonidazole ([F-18]-FMISO) is the most widely used positron emission tomography (PET) tracer for the imaging of tumor hypoxia. [F-18]-FMISO is a 2-nitroimidazole compound, which is reduced by nitroreductase enzymes in a hypoxic environment and trapped in hypoxic tumor cells. The hypoxia probe [F-18]-FMISO and the hypoxia marker pimonidazole are imidazole derivatives and they accumulate in similar regions in tumors (6,7). [F-18]-FMISO imaging of hypoxia may enable the optimization of radiotherapy planning and the prediction of radiotherapy outcome (8–10). Furthermore, hypoxia imaging using nitroimidazole has been used for patient selection for hypoxia-modifying treatments, including high-oxygen-content gas breathing and nitrometric radiation sensitizers (11,12).

Aside from oxygen concentration, biological characteristics related to hypoxia have also been reported to affect radiosensitivity and radiotherapy outcome (4,13,14). Thus, it is important to elucidate the relationship between [F-18]-FMISO distribution and biological characteristics. However, the biological characteristics of intratumoral [F-18]-FMISO distribution have not yet been fully investigated. Extensive studies have been carried out on gene/protein expressions in hypoxic regions. Genes inducing glucose metabolism are upregulated in a hypoxic environment by hypoxia-inducible factor-1 (HIF-1) transcription factor (15,16). HIF-1 target proteins include the glucose transporter-1 (Glut-1) and hexokinase-II (HK-II). HIF-1 and Glut-1 have been used as endogenous hypoxia markers to predict the response to radiotherapy (17). The expression of Glut-1 and the functional activity of HK-II correlate with glucose metabolism in malignant tumors (18,19). HIF-1 also upregulates genes inducing cellular proliferation (20) and knockdown of HIF-1α results in a decrease in cellular proliferation rate *in vivo*(16). We therefore compared the intratumoral [F-18]-FMISO distribution with Glut-1 and Ki-67 expression and with [C-14]2-fluoro-2-deoxy-D-glucose ([C-14]-FDG) distribution in a rat glioma model, in order to gain insight into the biological characteristics of intratumoral [F-18]-FMISO distribution that is relevant to radiotherapy planning.

## Materials and methods

### Animal studies

The experimental protocol was approved by the Laboratory Animal Care and Use Committee of Hokkaido University. Eight-week-old male Wistar King Aptekman/Hok (WKAH) rats (supplied by Japan SLC, Inc., Hamamatsu, Japan) were inoculated with a suspension of allogenic C6 rat glioma cells (2×10^6^ cells/0.2 ml) into the left calf muscle to generate a rat glioma model (21). The rats were allowed free access to water and laboratory chow until the day before the experiment. Nine days after the tumor inoculation, when the tumors had reached 1–2 cm in diameter, the rats were fasted overnight (n=5). Under diethyl ether anesthesia, the tail vein was injected with a mixture of 29–37 MBq of [F-18]-FMISO and 370–493 kBq of [C-14]-FDG. [F-18]-FMISO (specific activity, 45–70 MBq/nmol) was synthesized as previously described (22,23). Universally labeled [C-14]-FDG (specific activity, 11.1 GBq/mmol) in sterile saline was purchased from American Radiolabeled Chemicals, Inc. Eighty minutes after the injection of [F-18]-FMISO and [C-14]-FDG, the rats were anesthetized with pentobarbital (50 mq/kg body weight, intraperitoneally). Ten minutes after the injection, the rats were injected into the tail vein with pimonidazole (Hypoxyprobe-1; HPI Inc., Burlington, MA, USA) at a dose of 60 mq/kg body weight. Sixty minutes after the pimonidazole injection the animals were sacrificed and the tumors were quickly excised. The calf muscles were excised with the tumors. Each specimen was then sectioned to obtain two adjacent 3–5-mm slices. One of the two slices was embedded in Tissue-Tek medium (Sakura Finetechnical Co., Ltd.) and frozen in isopentane/dry ice for autoradiography (ARG) and immunohistochemical (IHC) analyses. The remaining slice was prepared as the formalin-fixed, paraffin-embedded specimens for IHC analyses of pimonidazole.

### Dual-tracer ARG

The frozen samples were cut into 20-*μ*m and 5-*μ*m adjacent sections with a CM3050-Cryostat (Leica Microsystems) at −20°C. Tumor sections (20 *μ*m and 5 *μ*m) were prepared for ARG. The radioactivity of [F-18]-FMISO was measured in 20-*μ*m sections. The radioactivity of [C-14]-FDG was measured in 5-*μ*m sections. [C-14]-FDG ARG images of 20-*μ*m sections were used for superimposing. Four 5-*μ*m tumor sections were prepared for IHC and hematoxylin-eosin (HE) staining. The 20-*μ*m and 5-*μ*m tumor sections were placed in a phosphor image plate cassette with a set of calibrated standards (24), and the specimens were exposed to phosphor imaging plates (Fuji Imaging Plate, Fuji Photo Film Co., Ltd.) overnight to detect the distribution of [F-18]-FMISO. During ARG exposure, a polypropylene film was set between the phosphor imaging plate and a set of tumor sections and calibrated standards to block the β-rays from [C-14]-FDG. Two days later, following the decay of [F-18]-FMISO, the same tumor sections and calibrated standards were exposed to a phosphor imaging plate for 14 days to detect the distribution of [C-14]-FDG. The HE staining of the sections was carried out to exclude necrotic/apoptotic regions in the regions of interest (ROIs) on the autoradiograms. The ARG images obtained were analyzed using a computerized imaging analysis system (BAS 5000 Bio-Imaging Analyzer; Fuji Photo Film Co., Ltd.). The ARG resolution of BAS 5000 was 25 *μ*m. The radioactivity in each ROI was expressed as the percentage activity of injected dose (ID) per gram of tissue following normalization to the animal’s weight (% ID/g/kg), with the hypothesis that the tissue density is 1 g/cm^3^(24,25).

### Immunohistochemistry

The experimental conditions of IHC were set to be identical using an automated staining system (Autostainer Plus, Dako) (26). The uptake of pimonidazole and the expression of Glut-1 and Ki-67 were examined in the frozen sections. Four adjacent tumor sections were used for pimonidazole, Glut-1, Ki-67, or HE staining. For pimonidazole staining, following rehydration, the slides were immersed in a citrate buffer solution (pH 6.0) and heated for 15 min at 121°C to retrieve the antigen. Subsequently, endogenous peroxidase activity was blocked for 10 min in 0.3% hydrogen peroxide. Thereafter, the slides were incubated with Hyproxyprobe-1 MAb1 (HPI Inc.) for 30 min at 37°C, and then with biotinconjugated F(ab’)2 for 15 min at 37°C. Following incubation with the antibodies, the bound antibody complex was visualized by incubation with streptavidin and 3,3-diaminobenzidine tetrahydrochloride. For Glut-1 and Ki-67 staining, the slides were immersed in a target retrieval solution (pH 9.0; Nichirei) and heated for 10 min at 95°C following rehydration. Endogenous peroxidase activity was then blocked for 10 min in 0.3% hydrogen peroxide. Thereafter, the slides for Glut-1 staining were incubated with anti-Glut-1 (Abcam) for 30 min at 37°C, and then with anti-rabbit immunoglobulins/biotinylated (Dako) for 30 min at 37°C. The slides for Ki-67 staining were incubated with an anti-Ki-67 antibody (Dako) for 30 min at 37°C, and then with anti-mouse immunoglobulins/biotinylated (Dako) for 30 min at 37°C. Following incubation with the antibodies, the bound antibody complex was visualized by incubation with streptavidin and 3,3-diaminobenzidine tetrahydrochloride. Tumor sections adjacent to those used for the immunostaining were stained with HE to exclude necrotic/apoptotic regions in ROIs on IHC images. IHC images were captured using a Biozero fluorescence microscope (BZ-8000; Keyence). The size of the IHC images was 2.32 pixels/*μ*m.

The uptake of pimonidazole and the expression of HK-II were studied in the formalin-fixed, paraffin-embedded tumor sections. Two adjacent tumor sections were used for pimonidazole or HK-II staining. Five-*μ*m adjacent sections were prepared using a Leika RM2265 microtome (Leica Microsystems). All paraffin-embedded sections were deparaffinized prior to antigen retrieval. For pimonidazole staining, the sections were stained similarly to the frozen sections, except in antigen retrieval. The antigen retrieval method for pimonidazole staining involved immersion in a citrate buffer solution (pH 6.0) and heating for 15 min at 121°C. For HK-II staining, the slides were immersed in a protease K solution (Dako) for 10 min at 37°C. The slides were then immersed in 0.3% hydrogen peroxide for 10 min. Thereafter, the slides were incubated with an anti-HK-II antibody (Chemicon International) for 30 min at 37°C, and then with anti-rabbit immunoglobulins/biotinylated (Dako) for 30 min at 37°C. Following antibody incubation, the bound antibody complex was visualized by incubation with streptavidin and 3,3-diaminobenzidine tetrahydrochloride (Dako).

### Image analysis

**Figure 1:**
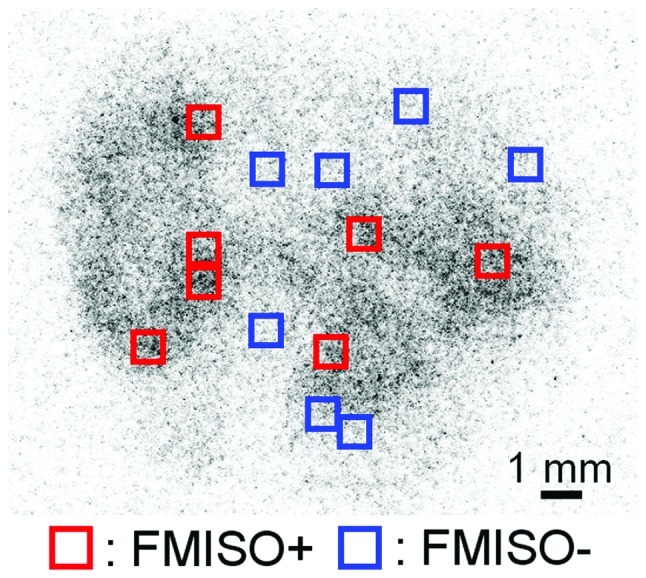
[F-18]-FMISO ARG image. ROIs were placed on [F-18]-FMISO ARG image to cover FMISO+ and FMISO−, except for large necrotic/apoptotic regions.

[F-18]-FMISO ARG images were compared with [C-14]-FDG ARG images and the IHC images of pimonidazole, Glut-1 and Ki-67 in the frozen sections. [F-18]-FMISO ARG images were divided into the regions of high [F-18]-FMISO uptake (FMISO+) and low [F-18]-FMISO uptake (FMISO−). The tumor-to-muscle (T/M) ratio of 4 was used as a cutoff value between FMISO+ and FMISO−, based on a previous report stating that the T/M ratio of [F-18]-FMISO uptake was 4.4±1.3 3 h following injection of [F-18]-FMISO to Walker 256 rat carcinosarcoma (27). The [F-18]-FMISO uptake level in the calf muscles around the tumor was measured. Three ROIs (0.04 mm^2^) were assigned to the muscle and the [F-18]-FMISO uptake level was determined in these ROIs. Large necrotic/apoptotic regions were excluded from the evaluation by referring to the sections stained in HE images. Initially, we set 0.16-mm^2^ areas in the entire FMISO+ and FMISO−. Then, we assigned random numbers to all the 0.16-mm^2^ areas. An individual not related to our study selected seven numbers from each random number set of FMISO+ and FMISO−. The areas that were assigned the selected numbers were determined as ROIs (Fig. 1). The [F-18]-FMISO uptake level in FMISO+ was significantly higher compared to FMISO− (FMISO+, 0.45±0.11% ID/g/kg; FMISO−, 0.18±0.04% ID/g/kg; P<0.001). The ROIs placed on the [C-14]-FDG ARG images were transferred from the [F-18]-FMISO ARG images using Fujifilm MultiGauge imaging software (Fujifilm Inc.). We manually colocalized ARG images with each other using MultiGauge. Initially, the ROIs placed on the [C-14]-FDG ARG images of 20-*μ*m sections were transferred from the [C-14]-FDG ARG images of 5-*μ*m sections by superimposing the margins of the tumor. Subsequently, the ROIs placed on the [F-18]-FMISO ARG images of 20-*μ*m sections were transferred from the [C-14]-FDG ARG images of 20-*μ*m sections by measuring the position of the phosphor imaging plates. IHC images were compared with [C-14]-FDG ARG images by Adobe Photoshop (Adobe Systems). The positions of the ROIs on the [C-14]-FDG ARG images were assigned to the corresponding positions of ROIs placed on the IHC images, by superimposing the margins of the tumor in the [C-14]-FDG ARG images with those in the IHC images. The percentage of positively stained areas (% positive) of pimonidazole and Glut-1 and the proliferation index of Ki-67 were quantified using ImageJ 1.41o software (National Institutes of Health). Thresholds for pimonidazole positivity and the proliferation index of Ki-67 were set above the background staining using a binary image.

The IHC images of pimonidazole in paraffin sections were compared with those of HK-II. The IHC images of pimonidazole were divided into pimonidazole-positive regions (Pimo+) and pimonidazole-negative regions (Pimo−). Pimo+ and Pimo− areas were defined as the areas containing only pimonidazole-positive and -negative cells, respectively. We compared the expression of HK-II between Pimo+ and Pimo−, rather than between FMISO+ and FMISO−, since the images of HK-II IHC staining of the frozen sections were not adequately stained for evaluation. Large necrotic/apoptotic regions were excluded from the evaluation by referring to images of the sections stained with HE. Twenty-five ROIs (0.04 mm^2^) were assigned from Pimo+ and Pimo− in a double-blind manner. The positions of the ROIs on IHC images of HK-II were assigned to the corresponding positions on IHC images of pimonidazole, using Adobe Photoshop (Adobe Systems). The % positive of pimonidazole and HK-II were quantified using ImageJ 1.41o software (National Institutes of Health). The threshold for HK-II positivity was set above the background staining using a binary image.

### Statistical analyses

In the analyses between FMISO+ and FMISO−, all values were averaged in seven ROIs from each tumor section. Subsequently, five values of FMISO+ and FMISO− per rat were statistically analyzed. All values are expressed as the means ± standard deviation. Statistical analyses were performed using a non-parametric Mann-Whitney U test to evaluate the significance of differences in values between FMISO+ and FMISO−.

In the analyses between Pimo+ and Pimo−, all values were averaged in 25 ROIs from each tumor section. Five values of Pimo+ and Pimo− per rat were then statistically analyzed. All values are expressed as the means ± standard deviation. Statistical analyses were performed using a non-parametric Mann-Whitney U test to evaluate the significance of differences in values between Pimo+ and Pimo−. P-value <0.05 was considered to indicate a statistically significant difference. The statistical program StatView 5.0 was used for data assessment.

## Results

### [F-18]-FMISO distribution in comparison with pimonidazole distribution

**Figure 2:**
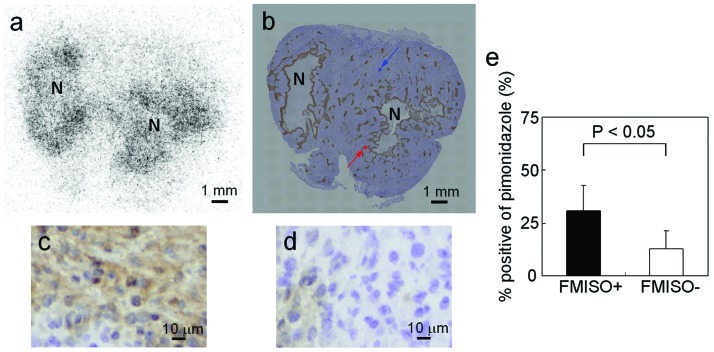
[F-18]-FMISO distribution in comparison with pimonidazole distribution. Representative images of (a) [F-18]-FMISO ARG and (b) pimonidazole IHC staining. Red and blue arrows denote the area of origin in panels c and d. Typical IHC stainings of pimonidazole in (c) FMISO+ and (d) FMISO−. (e) Pimonidazole uptake assessed by semiquantitative analysis in FMISO+ and FMISO−. N, necrotic/apoptotic regions.

Fig. 2a and b shows representative images of [F-18]-FMISO ARG and pimonidazole IHC staining of the whole tumor. The patterns of pimonidazole uptake were similar to those of [F-18]-FMISO uptake. The typical IHC stainings of pimonidazole in FMISO+ and FMISO− are shown in Fig. 2c and d, respectively. The intensity and extent of pimonidazole staining were markedly greater in FMISO+ compared to FMISO−. The results of semiquantitative analysis of pimonidazole uptake are summarized in Fig. 2e. The % positive of pimonidazole was significantly higher in FMISO+ compared to FMISO− (31±12% in FMISO+ and 13±9% in FMISO−; P<0.05).

### Glut-1 expression in comparison with [F-18]-FMISO distribution

**Figure 3:**
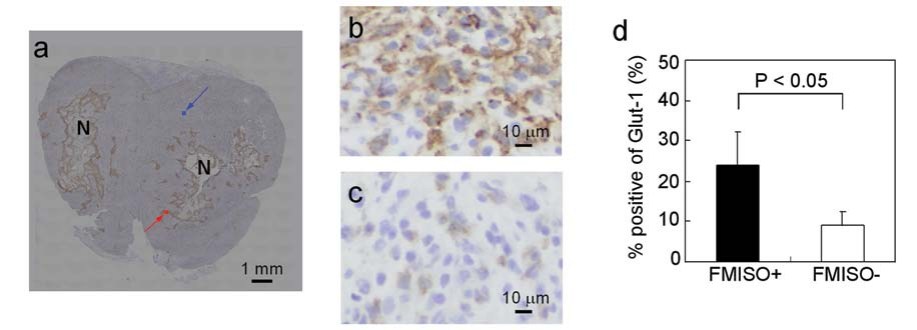
Glut-1 expression in comparison with [F-18]-FMISO distribution. (a) Representative image of Glut-1 IHC staining. Red and blue arrows denote the area of origin in panels b and c. Typical IHC stainings of Glut-1 in (b) FMISO+ and (c) FMISO−. (d) Glut-1 expression assessed by semiquantitative analysis in FMISO+ and FMISO−. N, necrotic/apoptotic regions.

Fig. 3a shows representative images of Glut-1 IHC staining of the whole tumor. The patterns of Glut-1 positively stained areas were similar to those of [F-18]-FMISO uptake (Fig. 2a). The typical IHC stainings of Glut-1 in FMISO+ and FMISO− are shown in Fig. 3b and c, respectively. The intensity and extent of Glut-1 staining were markedly greater in FMISO+ compared to FMISO−. The results of semiquantitative analysis of Glut-1 are summarized in Fig. 3d. The % positive of Glut-1 was significantly higher in FMISO+ compared to FMISO−(24±8% in FMISO+ and 9±4% in FMISO−, P<0.05).

### Ki-67 expression in comparison with [F-18]-FMISO distribution

**Figure 4:**
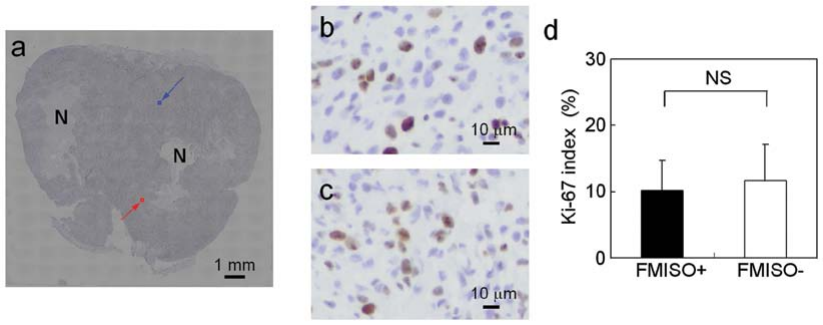
Ki-67 expression in comparison with [F-18]-FMISO distribution. (a) Representative image of Ki-67 IHC staining. Red and blue arrows denote the area of origin in panels b and c. Typical IHC stainings of Ki-67 in (b) FMISO+ and (c) FMISO−. (d) Ki-67 expression assessed by semiquantitative analysis in FMISO+ and FMISO−. N, necrotic/apoptotic regions.

Fig. 4a shows a representative Ki-67 IHC staining of the tumor. The typical IHC stainings of Ki-67 in FMISO+ and FMISO− are shown in Fig. 4b and c, respectively. Ki-67-positive cells were observed in both FMISO+ and FMISO−. The results of semiquantitative analysis of Ki-67 are summarized in Fig. 4d. There were no significant differences in Ki-67 index between FMISO+ and FMISO− (10±5% in FMISO+ and 12±5% in FMISO−; P = ns).

### [C-14]-FDG distribution in comparison with [F-18]-FMISO distribution

**Figure 5:**
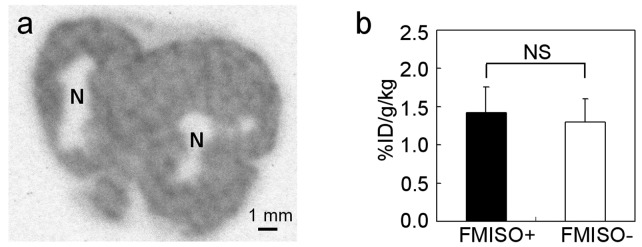
[C-14]-FDG distribution in comparison with [F-18]-FMISO distribution. (a) Representative image of [C-14]-FDG ARG. (b) [C-14]-FDG uptake assessed by semiquantitative analysis in FMISO+ and FMISO−. N, necrotic/apoptotic regions.

Fig. 5a shows a representative [C-14]-FDG ARG image of the tumor. [C-14]-FDG accumulation was observed in the entire tumor except in necrotic/apoptotic regions. The results of semiquantitative analysis of [C-14]-FDG accumulation based on ARG images are summarized in Fig. 5b. There were no significant differences in [C-14]-FDG uptake between FMISO+ and FMISO− (1.4±0.3% ID/g/kg in FMISO+ and 1.3±0.3% ID/g/kg in FMISO−; P = ns).

### HK-II expression in comparison with pimonidazole distribution

**Figure 6:**
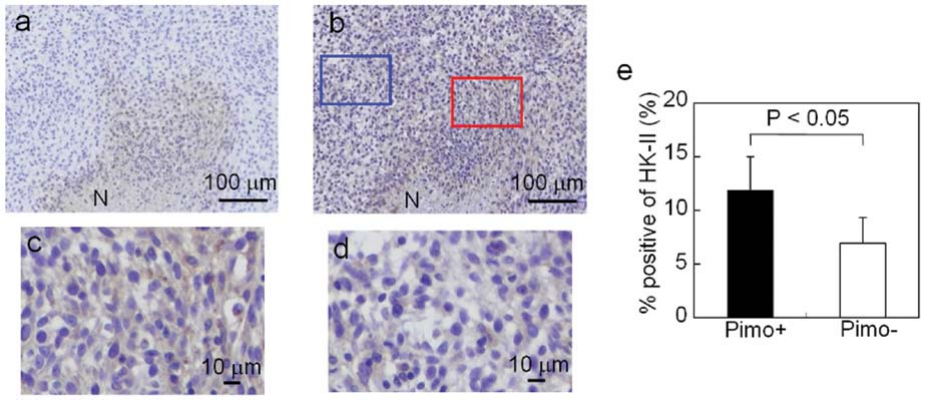
HK-II expression in comparison with pimonidazole distribution. Representative images of (a) pimonidazole and (b) HK-II IHC staining. Typical immunostaining of pimonidazole in (c) Pimo+ and (d) Pimo−. (e) HK-II expression assessed by semiquantitative analysis in Pimo+ and Pimo−. N, necrotic/apoptotic regions.

Fig. 6a shows representative images of pimonidazole IHC staining of the tumor. Fig. 6b shows representative images of HK-II IHC staining of the tumor. The staining pattern of pimonidazole-positive areas was similar to that of HK-II-positive areas. The typical IHC stainings of HK-II in Pimo+ and Pimo− are shown in Fig. 6c and d, respectively. The intensity and extent of HK-II staining were markedly greater in Pimo+ compared to Pimo−. The results of semiquantitative analysis of HK-II are summarized in Fig. 6e. The % positive of HK-II was significantly higher in Pimo+ compared to Pimo− (12±3% in Pimo+ and 7±2% in Pimo−; P<0.05).

## Discussion

In this study, the % positive of pimonidazole and Glut-1 were higher in FMISO+ than in FMISO−. However, there were no significant differences in Ki-67 index and [C-14]-FDG uptake between FMISO+ and FMISO−. These findings indicate that intratumoral [F-18]-FMISO distribution reflects tumor hypoxia and expression of the hypoxia-related gene product Glut-1; it does not, however, reflect tumor proliferation or glucose metabolism. Radiosensitivity depends on oxygen levels in tumor tissues (2). Hypoxia imaging using [F-18]-FMISO is therefore considered useful for patient selection prior to and during radiotherapy (8–10). Moreover, biological characteristics affect radiosensitivity and radiotherapy outcome (4,13,14). Therefore, [F-18]-FMISO may be used to identify hypoxic radioresistant regions among regions with similar proliferative activity and glucose metabolism activity. Such regions may be targets for dose escalation using IMRT. Our results may thus provide critical information on radiotherapy, since patients suffering from malignant glioma are generally treated by fractionated radiation therapy (28).

Our study indicated that [F-18]-FMISO distribution was similar to pimonidazole distribution and that Glut-1 expression level was higher in FMISO+. Since pimonidazole uptake is closely related to tumor hypoxia, pimonidazole has been used as an exogenous marker of radioresistance in clinical practice. Kaanders *et al* demonstrated that locoregional tumor control and disease-free survival were significantly reduced in patients who had head-and-neck tumors with high pimonidazole binding levels (29). These differences were not observed in the subgroup of patients undergoing accelerated radio-therapy combined with carbogen and nicotinamide (ARCON) treatment. This indicated that pimonidazole binding reflects hypoxic radiation resistance. While pimonidazole is used as the exogenous marker, Glut-1 has been used as an endogenous marker. Airley *et al* investigated the relationship between Glut-1 expression in cervical tumors and the prognosis following treatment of these tumors with radiotherapy (30). A high Glut-1 staining intensity in tumors indicated a shorter metastasis-free survival. This suggested that Glut-1 expression may be a potential marker of radioresistance. Therefore, the increases in pimonidazole uptake and Glut-1 expression level in FMISO+ suggest that tumor cells in FMISO+ may be more radioresistant compared to those in FMISO−.

Cellular proliferation and glucose metabolism are indicators of biological aggressiveness. Therefore, tumor areas with a high cellular proliferation or glucose metabolism may be an important target for radiotherapy, comparable with hypoxic tumor areas. Tumor hypoxia may correlate with cellular proliferation and glucose metabolism, since HIF-1 upregulates genes that induce cellular proliferation and glucose metabolism. We confirmed that the Glut-1 expression level was increased in FMISO+. Moreover, we discovered that the HK-II expression level was higher in high-pimonidazole-uptake regions (Pimo+) than in low-pimonidazole-uptake regions (Pimo−) (Fig. 6). However, we observed no significant differences in cellular proliferation and glucose metabolism between FMISO+ and FMISO−. Several studies have demonstrated discordant results regarding the correlation between hypoxia and cellular proliferation, or between hypoxia and glucose metabolism (4,5,31–36). The expressions of Glut-1 and HK-II are important factors that induce glucose metabolism (18,19). However, our data demonstrated that glucose metabolism in FMISO+ was not significantly enhanced, even when the expression levels of Glut-1 in FMISO+ and HK-II in Pimo+ were increased. There was also no notable increase in cellular proliferative activity. Therefore, it is hypothesized that factors other than Glut-1 and HK-II expression may predominantly affect glucose metabolism and cellular proliferation in FMISO+. For example, the delivery of glucose is reduced in hypoxic regions due to their long distance from blood vessels. The reduced glucose delivery may result in decreased glucose metabolism and cellular proliferation.

Riesterer *et al* demonstrated that [F-18]-FMISO distribution in tumors is similar to the distribution of Glut-1-positive regions in the mouse mammary tumor model (37), which is consistent with our results in the glioma rat model. Regarding the correlation between oxygen concentration and proliferative activity, the proliferative activity in hypoxic regions was decreased in several types of tumors (5,38). However, Evans *et al* reported that the hypoxia probe EF5-binding regions with a proximity to Ki-67-positive cells (approximately 50 *μ*m) were the 75.6% of all EF5-binding regions in human glioblastoma, suggesting that the majority of the hypoxic regions overlap with highly proliferative regions in human glioblastoma (4). Therefore, the correlation between oxygen concentration and proliferative activity is considered to differ among different types of tumors and this correlation in C6 glioma has not been fully investigated. We discovered that the proliferative activity in FMISO+ was not significantly different from that in FMISO− in C6 glioma. Regarding the correlation between oxygen concentration and glucose metabolism, hypoxia probe uptake in tumors was weakly or not correlated with glucose metabolism in several types of tumors (31,39,40). In C6 glioma, however, the correlation has not been elucidated. Our results in C6 glioma also demonstrated a weak or no correlation between [F-18]-FMISO distribution and glucose metabolism.

Although [F-18]-FMISO imaging may be useful for radiotherapy planning, several issues remain to be addressed. First, it is difficult to discriminate hypoxic from normoxic regions due to the low resolution of clinical PET. Furthermore, hypoxic regions in tumors were reported to be unstable (41). To optimize radiotherapy planning by [F-18]-FMISO imaging, [F-18]-FMISO−positive regions must match hypoxic regions at the time of radiation delivery.

There is an increasing interest in incorporating functional and molecular information into radiotherapy. Molecular imaging, including [F-18]-FDG and [F-18]-FMISO PET/CT, can provide such information (42). At present, radiotherapy planning using [F-18]-FDG and [F-18]-FMISO PET/CT is mostly conducted without detailed biological information regarding probe distribution. It is critical to elucidate the biology underlying molecular imaging. Our results provide useful insight into radiotherapy planning using [F-18]-FMISO PET, although further studies are required to determine the optimal use of such biological information in radiotherapy planning.

In conclusion, intratumoral [F-18]-FMISO distribution reflected tumor hypoxia and the expression of the hypoxia-related gene product Glut-1; it did not, however, reflect tumor proliferation or glucose metabolism. Our findings may help elucidate the biological characteristics of intratumoral [F-18]-FMISO distribution that are relevant to radiotherapy planning.
